# “Do You Hear What I Hear?” Speech and Voice Alterations in Hearing Loss: A Systematic Review

**DOI:** 10.3390/jcm14051428

**Published:** 2025-02-20

**Authors:** Arianna Di Stadio, Jake Sossamon, Pietro De Luca, Iole Indovina, Giovanni Motta, Massimo Ralli, Michael J. Brenner, Elliot M. Frohman, Gordon T. Plant

**Affiliations:** 1 Otolaryngology Department, Vanvitelli University, 80138 Naples, Italy; giovannimotta95@yahoo.it; 2 Medical University of South Carolina, Charleston, SC 29425, USA; sossamon@musc.edu; 3 Department of Otolaryngology, Fatebenefratelli-Isola Hospital, 00186 Rome, Italy; dr.dlp@hotmail.it; 4 Department of Systems Medicine, Centre for Space BioMedicine, University of Rome Tor Vergata, 00133 Rome, Italy; i.indovina@hsantalucia.it; 5 Laboratory of Neuromotor Physiology, IRCCS Foundation Santa Lucia, 00179 Rome, Italy; 6 International Medical University UNICAMILLUS, 00131 Rome, Italy; massimo.ralli@unicamillus.org; 7 Department of Otolaryngology—Head and Neck Surgery, University of Michigan Medical School, Ann Arbor, MI 48109, USA; 8 Neuroimmunology Laboratory of Professor Lawrence Steinman, Stanford University School of Medicine, Palo Alto, CA 94305, USA; elliotfrohman123@gmail.com; 9 Department of Brain Repair and Neurorehabilitation, University College London, London WC1E 6BT, UK; g.plant@ucl.ac.uk

**Keywords:** hearing loss, pitch, loudness, cochlear implant, auditory rehabilitation, voice modulation, compensatory mechanisms, age-related hearing loss, presbycusis

## Abstract

**Background**: Although hearing loss influences voice characteristics, such changes may be under-recognized during clinical consultations. This systematic review examines voice alterations in adults with post-lingual hearing loss, considering diagnostic and rehabilitative implications. **Methods**: A comprehensive search of PubMed, Scopus, and Google Scholar was conducted following PRISMA guidelines, targeting studies reporting quantitative data on vocal parameters in adults with sensorineural hearing loss. Exclusion criteria included pre-lingual hearing loss and non-English studies. Data extraction focused on pitch, loudness, and prosody, with study quality assessed using NIH tools. **Results:** Eleven case–control studies, involving 594 patients with sensorineural hearing loss and 326 control patients, were analyzed. Patients with untreated hearing loss exhibited elevated fundamental frequency, F_0_ (males: 158–169 Hz; females: 206–251 Hz) and loudness levels (males: 79–96 dB; females: 89–116 dB) compared to controls (F_0_—males: 75–150 Hz; females: 150–300 Hz; loudness—males: 30–70 dB; females: 40–68 dB). Alterations in jitter, shimmer, and maximum phonation time (MPT) contributed to the distinct “hearing loss voice”. Cochlear implants (CIs) and hearing aids improved vocal parameters, with CIs reducing F_0_ by approximately 12–15 Hz. Continuous hearing aid use normalized pitch and loudness within four months. Prosody alterations, such as monotone speech, were reported in long-term cases. In noisy environments, individuals with hearing loss exhibited exaggerated increases in pitch and loudness, indicative of compensatory mechanisms. **Conclusions:** Post-lingual hearing loss disrupts the central regulation of voice, altering pitch, loudness, and other vocal parameters. Recognizing these changes, particularly in noisy environments, could facilitate the early diagnosis and timely rehabilitation of hearing deficits, potentially mitigating associated risks of cognitive decline.

## 1. Introduction

Hearing loss is a pervasive but often underdiagnosed condition with significant consequences. Untreated hearing loss has been strongly associated with cognitive decline, dementia, and social isolation, making early detection and intervention critical. Despite its deleterious effects on quality of life and long-term health, hearing loss frequently remains unrecognized in clinical practice, in part due to the subtlety and variability of its early manifestations [1]. One underexplored avenue for early identification is through the analysis of voice and speech characteristics, which are known to change with hearing loss. These changes often go unnoticed during routine clinical consultations. Many neurological conditions manifest with voice or speech-related symptoms, such as dysarthria and dysphonia, which can complicate diagnosis and interpretation [2,3,4]. Paying close attention to voice characteristics, particularly in individuals with severe bilateral hearing loss (HL), can provide diagnostic clues about underlying auditory deficits [5]. Both the severity and duration of hearing loss have been shown to influence voice quality [6].

While it is well-recognized that hearing loss can alter voice, the characteristics of these changes in patients with post-lingual, acquired hearing loss remain poorly understood. Existing research highlights the influence of the severity and duration of hearing loss on voice quality, yet a systematic understanding of these relationships has not been established [6]. This gap in knowledge limits the ability of clinicians to leverage vocal changes as diagnostic clues for hearing loss, particularly in patients with severe bilateral hearing loss. Each human has distinct pitch, volume, and timbre that have potential to be altered in hearing loss [7]. The human ear easily detects changes in these parameters when the listener is paying attention to alterations. The pitch is a function of the fundamental frequency (F_0_) that is generated by vocal cord (VC) vibrations. Pitch is measured in Hertz (Hz), with high tones corresponding to high-frequency VC vibrations and low tones to low-frequency VC vibrations. Loudness, measured in decibel (dB), is the measure of voice volume and can vary depending on the context of communication and emotional states [8,9]. Timbre, which is shaped by the resonance structures above the larynx, arises from harmonic frequencies (F1 and F2) that are multiples of F_0_. Timbre contributes to the voice’s tonal quality [8]. Speech prosody refers to the rhythm and intonation patterns of speech; it allows speakers to convey meaning and emotional nuance, helping listeners distinguish between questions, statements, and subtler psychological cues [10].

While clinicians cannot directly perceive a patient’s subjective hearing loss, they can readily discern altered vocal characteristics. These signs of hearing loss, easily observable during clinical consultations, create an opportunity for clinicians to identify auditory deficits early. Recognizing and quantifying these changes could not only enhance detection but also facilitate timely rehabilitation, potentially mitigating the associated risks of cognitive decline and social isolation [1]. In this systematic review, we aim to address this gap by identifying changes in pitch and loudness among patients with post-lingual acquired hearing loss. Such insights can advance our understanding of how hearing loss alters vocal characteristics and promote early detection and intervention for hearing loss.

## 2. Materials and Methods

**Figure 1:**
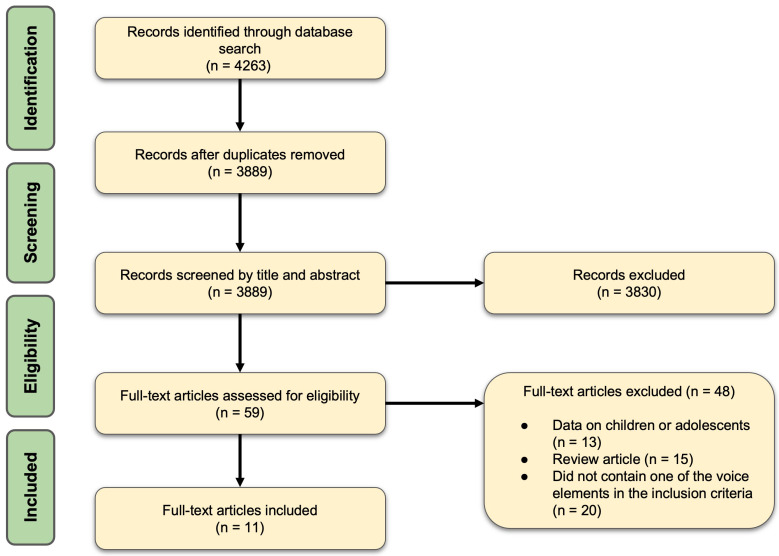
PRISMA Flow chart illustrating the process of our systematic review.

This study was conducted in accordance with the Preferred Reporting Items for Systematic Reviews and Meta-Analyses (PRISMA) guidelines (Figure 1). Ethical approval from the Institutional Review Board was not required for this systematic review.

### 2.1. Search Strategy

Two researchers (JS and PDL), under the supervision of ADS, performed a comprehensive literature search on PubMed, Scopus, and Google Scholar without geographical or time restrictions. The search incorporated a combination of MeSH terms and free-text keywords, such as “Voice and Hearing Loss”, “Speech and Hearing Loss”, “Speech and Deafness”, “Voice and Deafness”, “Hearing Loss and Vocal Characteristics”, “Communication and Hearing Loss”, and “Voice Characteristics and Hearing Loss”. Boolean operators (AND, OR) were applied strategically to refine the searches, ensuring sensitivity and specificity.

Abstracts of all extracted articles were independently screened, and duplicate entries were identified and systematically removed using reference management software. Articles were then cross-checked for consistency, and disagreements on the inclusion or exclusion of studies were resolved through structured discussions and iterative consensus meetings involving all three researchers (JS, PDL, and ADS). To ensure methodological transparency and reproducibility, the systematic review adhered strictly to the PRISMA guidelines, with a PRISMA flow diagram developed to track the selection process. Reference lists of included studies were manually reviewed to identify additional relevant articles that may have been overlooked in the database searches.

### 2.2. Study Selection Criteria

The inclusion and exclusion criteria were pre-specified to ensure a rigorous selection process. Pre-lingual cases were excluded to avoid confounding effects from developmental factors, such as delayed auditory or speech rehabilitation, which can result in persistent voice alterations unrelated to the acquired loss of hearing.

Inclusion Criteria:

- Studies providing quantitative data on at least one of the following parameters: fundamental frequency (F_0_)/pitch, loudness, or prosody in patients with sensorineural hearing loss.
- Studies published in peer-reviewed journals.
- Studies involving adult patients with post-lingual sensorineural hearing loss.
- Articles published in English.

Exclusion Criteria:

- Studies involving children or adolescents under 18 years old.
- Studies focusing exclusively on pre-lingual hearing loss.
- Studies lacking data on the specified vocal parameters (F_0_/pitch, loudness, or prosody).
- Non-peer-reviewed articles, conference abstracts, or opinion pieces.

### 2.3. Data Extraction

Data extraction was independently performed by two researchers (ADS and PDL) to ensure accuracy and minimize bias. A structured data extraction form was developed and piloted before use. The following key information was systematically recorded for each study: author name, year of publication, study design, country of origin, sample size, patient demographics, hearing loss characteristics, specific vocal and speech parameters assessed (including pitch, loudness, or prosody), and any additional outcomes related to voice or communication characteristics. To maintain data integrity, all extracted information was cross-verified by the supervising investigator (ADS) in biweekly review sessions. Any discrepancies identified during data extraction were resolved through discussion or, if necessary, consultation with an external expert in systematic reviews.

The extracted data were organized in a detailed spreadsheet for quantitative synthesis and qualitative analysis. This approach facilitated the identification of trends, gaps, and potential sources of bias across the included studies.

### 2.4. Risk of Bias Assessment

The National Institutes of Health (NIH) quality assessment tools for case–control studies were used to assess the risk of bias, as these checklists are designed for various study designs (National Heart, Lung, and Blood Institute. Study Quality Assessment Tools. (2014). Available at: https://www.nhlbi.nih.gov/health-topics/study-quality-assessment-tools, accessed on 14 November 2024). Studies were categorized as poor, fair, or good based on their quality, with special consideration given to phonological analyses. Incomplete phonological data were considered a source of bias, and studies were rated fair even if they were unbiased and fully described. Two authors (ADS and PDL) independently scored each article, and disagreements were resolved by consensus. The results are summarized in Table 1.

## 3. Results

**Figure 2:**
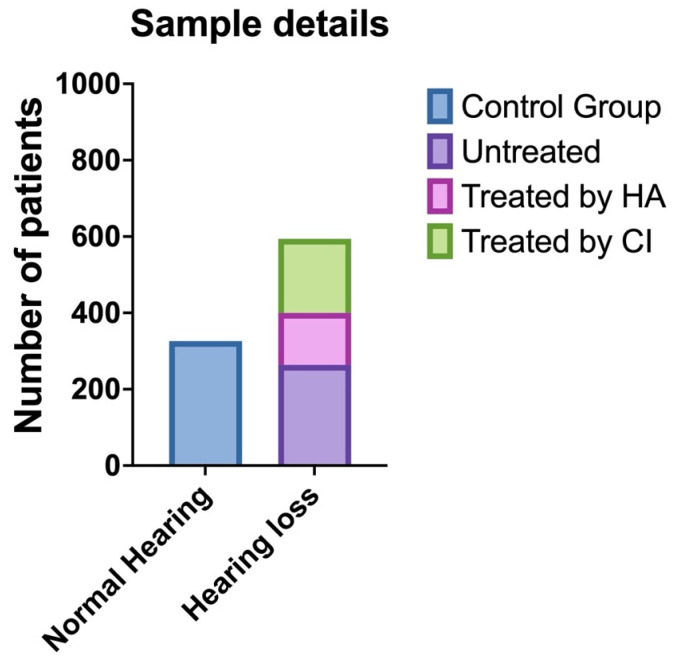
The graph shows the sample distribution between control (normal hearing) and patients with hearing loss. The latter are represented with different colors based on the treatment used/not used (HA = hearing aid; CI = cochlear implant).

A total of 11 articles were included in this study (Figure 1) [5,6,11,12,13,14,15,16,17,18,19]. The studies included 594 patients with sensorineural hearing loss who were compared with 326 control subjects without hearing loss (Figure 2). Four articles (36.4%) specifically stated that the studies were conducted on individuals with post-lingual auditory deficits. In the remaining cases (63.6%), the patients were adults with sensorineural hearing loss. The cause of sensorineural hearing loss was not defined in any of the studies.

All included studies were prospective observational case–control studies. Three were conducted in Italy (27.7%), while the others were conducted in the United States, Australia, Taiwan, Brazil, Iran, Turkey, Sweden, and Germany (Table 2).

In five studies (45.4%), voice characteristics were analyzed only at baseline without the use of hearing aids. Four studies (36.3%) examined the voice at baseline and after cochlear implant rehabilitation, and two studies (18.3%) assessed the voice at baseline and after using hearing aids (Table 2 and Figure 2).

In one article (9%), data on pitch, loudness, and prosody were available. Three papers (27%) reported both pitch and loudness, while the remaining seven articles (64%) provided data only on pitch (Table 2).

We summarized the results of the quality assessment performed on the studies included in this systematic review in Table 2. Five studies (45.5%) received quality rating consensus of good quality, while the remaining six (54.5%) received a rating of fair.

F_0_ was consistently increased in the presence of hearing loss; however, the use of hearing aids (cochlear implant or traditional hearing prosthesis) allowed for a reduction in F_0_. The voice loudness of people with hearing loss also increased when compared to loudness in individuals with normal hearing (data reported in four studies only). Prosody, reported in only one study, was reduced in patients with hearing loss when compared to individuals with normal hearing.

## 4. Discussion

The results of this systematic review reveal that patients with moderate to severe hearing loss exhibit higher pitch (F_0_) and louder volume compared to gender-matched controls. Furthermore, the extent of change correlated with the severity of sensory auditory loss. In addition, other vocal parameters, such as jitter, shimmer, soft phonation index, and maximum phonation time, were also altered in individuals with hearing loss. Together, these findings define a distinct “hearing loss voice”. These parameters can be detected during speaking; jitter and shimmer are perceived as roughness, breathiness, or hoarseness in a patient’s voice. Soft phonation index and time are also perceived as slight roughness and reduced time of phonation (<10 s).

### 4.1. Methodological Variations and Its Implications

The studies had heterogeneity in the reporting of hearing impairment, the vocal parameters studied, and the methodologies used to assess them. In 83.3% of the studies (Table 2), researchers employed ambulatory phonation monitors, whereas the remaining researchers used the Multidimensional Voice Program (MDVP) [20]. MDVP is a software tool that analyzes several vocal characteristics beyond F_0_, including jitter, shimmer, noise-to-harmonic ratio (NHR), soft phonation index (SPI), degree of voice break (DVB), degree of voicelessness (DUV), and peak amplitude variation (vAm) (Table 3).

**Figure 3:**
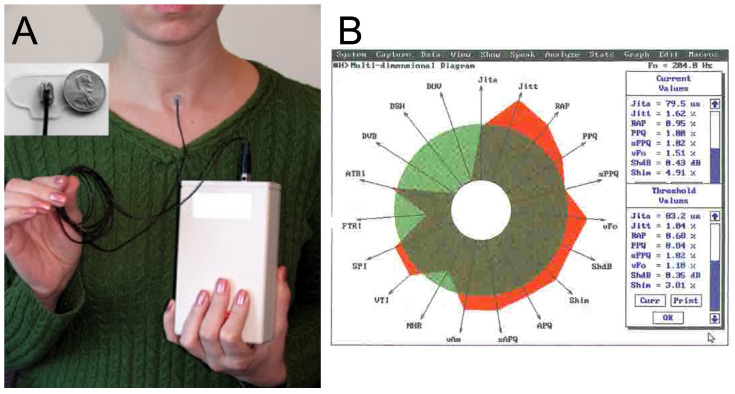
(**A**) The image shows the portable device to analyze the voice characteristics used in some of the studies included in the review. (**B**) The graph shows how the results of MDPV visually appear to the operator during the analysis of voice.

While MDVP provides a more comprehensive analysis of voice characteristics, allowing researchers to define a clearer “hearing loss voice”, its use is limited in clinical settings. Additionally, MDVP assessments are typically performed for a brief period, not capturing the patient’s voice in everyday conditions. In contrast, ambulatory phonation monitors [21] are simpler to use. When used as portable devices [22], they have the advantage of being wearable throughout the day, enabling voice analysis in real-life contexts. However, these devices are more limited in the range of vocal characteristics they can assess compared to MDVP (Figure 3).

Mora et al. [5] used MDVP to compare the voices of people with hearing loss to those of healthy individuals. They found that, in addition to a rise in fundamental frequency (137.2 Hz vs. 120.0 Hz), there were significant differences in jitter (1.93% vs. 0.67%), shimmer (6.67% vs. 3.81%), NHR (0.19 vs. 0.10), SPI (12.9 vs. 8.76), DVB (2.12% vs. 0.01%), DUV (9.53% vs. 0.51%), and vAm (23.12% vs. 12.06%). All these parameters tended to worsen as the degree of hearing loss increased.

Akil et al. [6] compared adults with normal auditory thresholds to those with mild-to-moderate bilateral sensorineural hearing loss (average: 25–60 dB) and moderate-to-profound sensorineural hearing loss (average: 60–90 dB). This study analyzed males and females separately and found significant differences in F_0_, variable F_0_ (vF_0_), absolute jitter, shimmer, SPI, and maximum phonation time (MPT) between healthy individuals and those with hearing loss. Men with hearing deficits exhibited the most significant changes in the vocal parameters studied. Individuals with mild-to-moderate hearing loss tended to become higher-pitched (acute F_0_) and louder. People with hearing loss exhibit escalated voice volume, with roughness, breathiness, or hoarseness of the voice. They also present a reduced time of phonation that is generally under 10 s that is punctuated by the need to stop and breathe before the resumption of speech.

### 4.2. Association of Interventions with Voice Characteristics

Studies of patients with hearing loss who were treated with cochlear implants (CIs) demonstrated improvements in F_0_ (reduced) and loudness (reduced) when the device was activated, compared to when it was switched off [18]. CI have a receiver and an electrode that through electronic stimulation within the cochlea facilitates listening and the accuracy of sound interpretation. The stimulation of the auditory cortex and laryngeal motor cortex [23] allows for effective and accurate sequential timing pattern commands to the larynx and vocal cords. In patients treated by CI, the voice exhibited can be indistinguishable from normal [18].

In patients with age-related moderate-to-severe hearing loss, using hearing aids continuously for at least four months resulted in reductions in both pitch and loudness, indicating that voice parameters can return to normal with proper prosthetic use [19]. The central control of vocal pitch and volume is complex and heavily influenced by auditory function, highlighting the interdependence of sensory input and motor output within and across sensory channels. This process involves interactions between diverse sensory modalities, culminating in sensory–motor integration. Beyond hearing and listening, such integration shapes our overall sensory experience and can give rise to synesthesia through the higher-order merging of information from distinct but interconnected sensory inputs.

### 4.3. Neurological Mechanisms Underlying Vocal Adjustments

**Figure 4:**
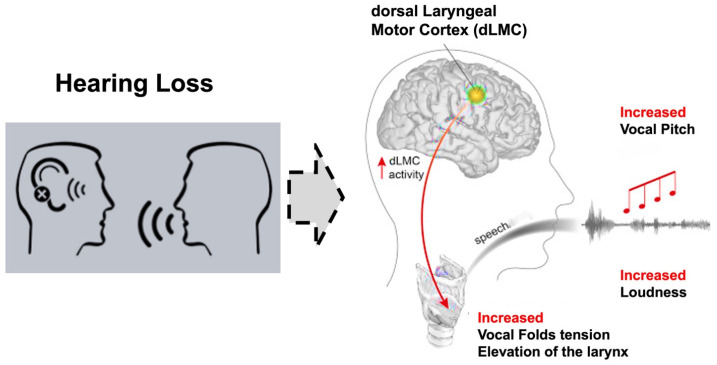
The drawing summarizes the mechanism that causes the increase in loudness and pitch in patients with hearing loss. We use the term “hearing loss voice” to summarize a high-pitch and high-volume voice. The image was modified from the original [23].

Dichter et al. used high-density cortical recordings from the human brain to investigate the encoding of vocal pitch during natural speech [23]. They found that neural populations in the bilateral dorsal laryngeal motor cortex (dLMC) selectively encoded produced pitch but not non-laryngeal articulatory movements. The dLMC controlled short pitch accents to express prosodic emphasis on words within sentences [23]. Speaking and listening require sensorimotor integration. The impulse to speak is initiated first, followed by listening, with pitch adjustments occurring during conversation. In individuals with hearing impairment, reduced auditory input likely disrupts this balance, contributing to an increase in pitch [24] (Figure 4).

### 4.4. Clinical Relevance and Role for Early Intervention

Patients with hearing loss tend to speak with a louder voice and exhibit escalated high-pitch intonation (high F_0_/pitch). In noisy environments, both parameters increase further [13]. These changes can be tested during clinical consultations by introducing noise (such as crumpling paper or tapping a pen on a desk) while asking questions to observe if the patient raises the volume and/or pitch in response. Generally, people without hearing deficits tend to maintain the same volume, whereas people with hearing loss rapidly transition to augmented speech volume and pitch.

In addition, due to the diminished ability of the auditory system to detect sound frequency distributions over time in hearing loss [25], patients with longstanding hearing loss may lose prosody [26] and develop a monotone voice. Therefore, earlier identification and treatment of hearing loss can produce disease modifying effects. While the diminished capacity of the auditory system to detect and effectively parse sound frequencies can result in the loss of prosody (i.e., the melodic aspects of speech) and a monotone voice, auditory rehabilitation could potentially prevent, delay, or even partly reverse these changes.

### 4.5. Limitations and Future Directions

This review has limitations relating to the limited evidence base and limited standardization of data reporting. All included studies were either case–control or observational studies. Randomized clinical trials evaluating the effect of hearing interventions on voice characteristics in individuals with hearing impairment would clarify causality and potential effects on cognitive performance.

Future research should prioritize longitudinal studies to better understand the progression of vocal changes in hearing loss and the potential for recovery with timely intervention. Innovative tools, such as machine learning algorithms and wearable technologies, could be developed to identify subtle vocal changes as early markers of auditory decline, enabling earlier diagnosis and intervention. These findings underscore the critical need for clinicians, researchers, and policymakers to work together in raising awareness of the vocal signs of hearing loss and integrating routine voice assessments into clinical practice. By doing so, we can ensure that individuals receive interventions sooner, preventing long-term complications and improving overall quality of life.

## 5. Conclusions

Hearing loss is associated with distinct changes to voice such as increased pitch and loudness, which are often compensatory mechanisms for impaired auditory feedback. These alterations become more pronounced as the severity and duration of hearing loss increase. Despite their diagnostic potential, these vocal changes are under-recognized during clinical consultations. The early identification of the “hearing loss voice” through attentive listening could provide a simple yet effective screening tool for clinicians. Such observations not only facilitate timely diagnosis and rehabilitation but also offer an opportunity to mitigate the broader cognitive and social consequences of untreated hearing loss [27]. Moreover, because untreated hearing loss is associated with neuroinflammation and neurodegeneration, the proactive use of hearing aids might slow or prevent such changes [28].

## Figures and Tables

**Table 1:** Results of the quality assessment performed on the included papers.

References	Design of the Study	Overall Quality Rating Consensus
Higgins [11]	Prospective case–control	Good
Weatherley et al. [12]	Prospective case–control	Fair
Lee [13]	Prospective case–control	Fair
Ubrig et al. [14]	Prospective case–control	Good
Akil et al. [6]	Prospective case–control	Fair
Hengen et al. [15]	Prospective case–control	Good
Zamani et al. [16]	Prospective case–control	Fair
Aria-Vergara et al. [17]	Prospective case–control	Good
Albera et al. [18]	Prospective case–control	Fair
Cardella et al. [19]	Prospective study	Fair
Mora et al. [4]	Prospective case–control	Good

**Table 2:** The table shows the articles included in the systematic review and the voice findings that were available to be analyzed.

Author, Year, Country	Study Type	Sample Size	Sample Characteristic (Age and Sex)	Affected	Control *	Treatment (Yes/No, Type of Hearing Treatment)	Voice Characteristic			Method of Voice Analysis
							*Pitch/F*_0_ *(Hz)*	*Loudness (dB)*	*Prosody (rhytm)*	
Higgins, 1994, United States [11]	POCCS	11	4 M, 7 F; mean age 42 yr	11	5	None	Females, 223.8 ± 53.2; Males, 158.5 ± 22.6			APM
Weatherley et al., 1997, Australia [12]	POCCS	40	gender n/a; mean age 74.5 yr	40	21	None	Females /a/ 189.68	Maximum loudness level 96.44		APM
							Females /i/ 211.72			
							Females /u/ 205.78			
							Males /a/ 127.46			
							Males /i/ 135.25			
							Males /u/ 135.02			
Lee, 2012, Taiwan [13]	POCCS	23	17 M, 6 F; mean age 66 yr	23	14	None	vF_0_% 2.71 ± 2.37	79.0 ± 7.7 SNHL vs. 83.9 ± 7.8 control		APM
Ubrig et al., 2010, Brazil [14]	POCCS	40 postlingual	20 M, 20 F; mean age 44.5 yr	40	12	CI	Male pre-CI, 154.2 ± 54.52; Male post-CI, 148.1 ± 58.18; Female pre-CI, 206.1 ± 43.01; Female post-CI, 199.9 ± 39.26			APM
Akil et al., 2017, Turkey [6]	POCCS	50	24 M, 26 F;	50	20	None	141 ± 24			APM
Hengen et al., 2018, Sweden [15]	POCCS	110 non-HA users + 110 HA users	non-HA users, 70 M, 40 F, mean age 70 yr/HA users, 59 M, 51F; mean age 74 yr	220	70	HA + none	Male non-HA users, 142.1 ± 28.2			APM
							Male HA users, 140.6 ± 31.8			
							Female non-HA users, 193.8 ± 29.1			
							Female HA users, 188.9 ± 26.5			
Zamani et al., 2021, Iran [16]	POCCS	48 post-lingual	17 M, 31 F; mean age 36.5 yr	48	50	CI	1st-on 166.2 ± 26.1			APM
							Off 169.2 ± 28.4			
							2nd on 166.1 ± 27.9			
Aria-Vergara et al., 2022, Germany [17]	POCCS	74 post-lingual	37 M, 27 F: mean age 66 yr	74	72	CI	134 ± 26	Mean SPL 69	Mean Std Voc (ms) 96	APM
								Mean Std SPL 15	Mean Std Con (ms) 79	
Albera et al., 2022, Italy [18]	POCCS	32, 16 prelingual/16 postlingual	16 M, 16 F; mean age 49.7 yr	32	32	CI	Male, CI off 156.5 ± 40	CI off 82.5 ± 11 dB		APM
							Male, CI on 150.8 ± 42	CI on 80.9 ± 13 dB		
							Female, CI off 251.2 ± 54			
							Female, CI on 218.4 ± 52			
Cardella, 2023, Italy [19]	POCCS	26	18 M, 8 F; mean age 71 yr	26	0	HA	Males before HA 130.1 ± 10.9			APM
							Males after HA 132.8 ± 8.8			
							Female before HA 212.4 ± 20.1			
							Female after HA197.1 ± 21.1			
Mora et al., 2012 [5]	POCCS	30 with HL; 30 healthy	60 M; 30 HL (35–53) and 30 control (38–51)	30	30	None	Male aftected 137 vs. 120 Control			MDVP

POCCS: prospective observational case–control study; CI: cochlear implant; HA: hearing aids; APM: ambulatory phonation monitor; MDVP: Multimensional Voice Program; HZ: hertz; dB: decibel; Std: standard deviation. *: including untreated patients in case of treatment vs. untreated patient studies.

**Table 3:** The definition of the relevant voice characteristics that are analyzed by MDVP.

***Jitter***	Measures cycle-to-cycle frequency variations, where cycle means opening and closure of vocal folds
***Shimmer***	Measures amplitude of sound wave variation
***Soft phonation index***	Measures approximation of vocal folds. High values correlate with incomplete vocal fold adduction
***Maximum phonation time***	The maximum amount of time a person can sustain phonation of “ah” is timed (typical range of 15 to 25 s in women and 25 to 35 s in men

## Data Availability

Data are available upon reasonable request to the corresponding authors.

## Abbreviations

The following abbreviations are used in this manuscript:

F_0_	Fundamental frequency
PRISMA	Preferred Reporting Items for Systematic Reviews and Meta-Analyses
MDVP	Multidimensional Voice Program
CI	Cochlear implant
NHR	Noise-to-harmonic ratio
SPI	Soft phonation index
DUV	Degree of voicelessness
DVB	Degree of voice break
vAm	Peak amplitude variation
vF_0_	Variable F_0_
MPT	Maximum phonation time
